# An empirical investigation of trust in AI in a Chinese petrochemical enterprise based on institutional theory

**DOI:** 10.1038/s41598-021-92904-7

**Published:** 2021-06-30

**Authors:** Jia Li, Yiwen Zhou, Junping Yao, Xuan Liu

**Affiliations:** 1 School of Business, East China University of Science and Technology, Shanghai, 200237 China; 2 Xi’an Research Institute of High-Tech, Xi’an, 710025 China

**Keywords:** Computer science, Mechanical engineering

## Abstract

Despite its considerable potential in the manufacturing industry, the application of artificial intelligence (AI) in the industry still faces the challenge of insufficient trust. Since AI is a black box with operations that ordinary users have difficulty understanding, users in organizations rely on institutional cues to make decisions about their trust in AI. Therefore, this study investigates trust in AI in the manufacturing industry from an institutional perspective. We identify three institutional dimensions from institutional theory and conceptualize them as management commitment (regulative dimension at the organizational level), authoritarian leadership (normative dimension at the group level), and trust in the AI promoter (cognitive dimension at the individual level). We hypothesize that all three institutional dimensions have positive effects on trust in AI. In addition, we propose hypotheses regarding the moderating effects of AI self-efficacy on these three institutional dimensions. A survey was conducted in a large petrochemical enterprise in eastern China just after the company had launched an AI-based diagnostics system for fault detection and isolation in process equipment service. The results indicate that management commitment, authoritarian leadership, and trust in the AI promoter are all positively related to trust in AI. Moreover, the effect of management commitment and trust in the AI promoter are strengthened when users have high AI self-efficacy. The findings of this study provide suggestions for academics and managers with respect to promoting users’ trust in AI in the manufacturing industry.

## Introduction

Artificial intelligence (AI) is increasingly popular. In addition to common applications in everyday life, such as facial recognition, autopilots, chatbots, and personalized recommendations, AI also has great potential in the manufacturing industry^1^. For example, AI can use the big data in the factory to improve the efficiency of the production process and reduce energy consumption^2^. AI can also use the data collected by Internet of Things (IoT) sensors to predict the failure of devices^3^. A typical AI based predictive maintenance can reduce annual maintenance costs by 10%, unplanned downtime by 25% and inspection costs by 25%^3^.

Despite its considerable potential in the manufacturing industry, the application of AI in companies still faces the challenge of insufficient trust. A recent survey shows that 42% of people lack basic trust in AI, and 49% can't even name an AI product they can trust^4^. In fact, people trust human experts more than AI, even if the human experts’ judgments are wrong^5^. If we want AI to really bring benefits to the manufacturing industry, we must find a way to earn human trust in it. Therefore, it is relevant to understand what prompts trust in AI in manufacturing companies.

Traditionally, to trust something, users must first be able to understand it and predict its behavior. That is, we cannot trust what we do not understand. However, the black box nature of AI makes it very difficult for users to understand it. For example, deep learning algorithms are becoming so complex that even their creators do not understand how they work. This complexity makes trust in AI very difficult because people must depend on other superficial cues to make trust decisions. In the individual context, such cues may include anthropomorphism^6^, voice consistency^7^, relationship type^8^, and timeliness in responding^9^ to AI. In the organizational context, trust in AI is subject to cues from the institutional environment. According to institutional theory, organizational and individual behavior are influenced by regulative, cognitive and normative institutional dimensions. Since AI systems are usually introduced by managers and promoted by key promoters, attitudes from top managers, group leaders and AI promoters should exert some influence on users’ trust in AI. Therefore, institutional theory is the appropriate theoretical lens through which to understand initial trust in AI within manufacturing companies. Accordingly, the first research question is as follows:

RQ1: How can regulative, normative and cognitive institutional dimensions influence user trust in AI in the manufacturing industry?

Users with different levels of AI self-efficacy also have different understandings of AI, which may further influence the impact of institutional elements on trust in AI. Such impact occurs because users with higher AI self-efficacy can perceive greater benefit to the company from using AI and fewer challenges (e.g., learning cost, potential interference in daily work) posed by AI for the individual. Accordingly, the second research question is as follows:

RQ2: Does AI self-efficacy moderate the effect of institutional dimensions on trust in AI in the manufacturing industry? If yes, how?

To answer these two research questions, this study proposes a research model based on institutional theory. Management commitment (regulative dimension at the organizational level), authoritarian leadership (normative dimension at the group level) and trust in the AI promoter (cognitive dimension at the individual level) are hypothesized to have a positive relationship with trust in AI. In addition, AI self-efficacy is hypothesized to positively moderate (strengthen) the impact of these three institutional dimensions. A field survey was conducted to test the proposed research model.

## Literature review

### Trust in AI

Trust in AI has received considerable attention in recent years. The antecedents of trust in AI are summarized and shown in Table 1. The categories of variables that may influence trust in AI include machine performance (e.g., machine capabilities^10^ or response quality/timeliness^9^), transparency (e.g., causability^11^, explainability^11,12^), representation (e.g., humanness^6^, facial features^13^, dynamic features^13^, emotional expressions^13^, virtual agents^14^), voice (e.g., voice consistent^7^ and perceived voice personality^15^), interaction (e.g., interaction quality^16^, consumer-chatbot relationship type^8^, reciprocal self-disclosure^17^, human-in-the-loop^18^), emotion (e.g., attachment style^19^), and user personal traits (e.g., big five personality characteristics^20^). Related studies also cover a wide range of contexts, including human-robot interaction^10,13^, conversational assistants^9,15,16^, recommendation systems^11^, medical computer vision^12^, speech recognition systems^14^, in-vehicle assistants^7^, and private or public services^6,18^.

**Table 1:** Studies on the antecedents of trust in AI.

Category	Context	Key variables	Source
Performance	Human-robot interaction	Machine intelligence (i.e., its capabilities), environmental factors	^10^
	AI chatbots in the public sector	Response quality, timeliness in responding	^9^
Transparency	News recommendation systems	Causability, explainability	^11^
	Medical computer Vision	Explainability	^12^
Representation	AI applications in service contexts	Anthropomorphism (humanness)	^6^
	Social robots	Static facial features, dynamic features, their combinations, and related emotional expressions	^13^
	Speech recognition systems	Virtual agents, XAI interaction design	^14^
Voice	In-vehicle assistants	Voice consistent	^7^
	Smart speakers	Perceived voice personality	^15^
Interaction	Voice assistant systems	Interaction quality	^16^
	AI-enabled chatbots	Consumer-chatbot relationship type (virtual assistantship versus virtual friendship)	^8^
	Conversational assistant	Reciprocal self-disclosure	^17^
	Decision aid utilized in the delivery of public services	The assurance that “humans are still in the decision loop”	^18^
Emotion	Supposed scenario: self-driving vehicles/autopilot, medical diagnostic aids, and personal relationship aids	Attachment style (attachment anxiety, attachment security)	^19^
Personal trait	An online trust game	Big five personality characteristics (e.g., openness to experience, conscientiousness)	^20^

As shown in Table 1, existing studies on trust in AI mainly focus on the individual context. That is, the decision of trust in AI is entirely made by individuals. However, research on how users trust AI in the organizational context (e.g., in a manufacturing company) is still lacking. In the organizational context, the decision of trust in AI is not completely personal. Users must consider the institutional influences of the company, the leader or peers before they make the final trust decision. Therefore, we will fill the gap regarding trust in AI in the organizational context by considering institutional influences in this study.

### Trust in organizational context

It is well known that trust is tightly related to many organization performance indicators such as organizational citizenship behavior^21^, policy compliance behavior^22^, turnover intentions^23^ and organizational performance^24^. Therefore, how to achieve a high level of organizational trust has become a very important research question. The antecedents of trust in the organization are summarized and shown in Table 2.

**Table 2:** Studies on the antecedents of trust in organizational context.

Category	Dependent variable(s)	Independent variable(s)	Source
Trust in organization	Trust in company	Interpretation of contract violations	^25^
	Employee’s trust in the organization	Organizational ethical climates (benevolent, principled and egoistic)	^26^
Trust in other organizations	Trust in other organizations in the supply chain	Information technology integration	^27^
	Trust in a cloud provider organization	Cloud service provider/platform provider reputation, institution based trust (competence, goodwill, integrity, reliability)	^28^
Trust in people in the organization	Employee trust in leaders	Transactional and transformational leadership behaviors	^29^
	Trust in top leaders	The relationships individuals have with their direct leaders	^30^
	Trust in organization stakeholders	Organizational transparency	^31^
Trust in IT artifacts in the organization	Initial trust in a national identity system	Organizational situational normality base factors	^32^
	Individual’s level of trust in the Human resource information systems	Organizational trust, organizational community, organizational culture, socialization	^33^
	Users trust in mobile commerce technologies	System quality, culture	^34^
	Trust in AI services	Supplier's declarations of conformity	^35^

The first category of trust focuses on employees' trust in the organization^25,26^. In two recent studies, both interpretation of contract violations^25^ and organizational ethical climates (benevolent, principled and egoistic)^26^ are used to explain the employees' trust in the organization. The second category of trust focuses on trust in other organizations^27,28^. In one study, information technology integration was found to promote trust among organizations in the supply chain^27^. In another study, service provider/platform provider reputation and institution based trust (competence, goodwill, integrity, reliability) were found to be positively related to trust in a cloud provider organization^28^. The third category of trust focuses on trust in people in the organization^29–31^. The transactional and transformational leadership behaviors^29^, the relationships individuals have with their direct leaders^30^, and organizational transparency^31^ are all possible antecedents of trust in leaders and key stakeholders in the organization. The last category of trust focuses on trust in IT artifacts in the organization. The organizational situational normality base factors^32^, organizational culture^33,34^, system quality^34^, supplier's declarations of conformity^35^ are identified as possible explanatory variables for trust in IT artifacts in the organization.

As shown in Table 2, institutional theory is seldom used to explain trust in the organizational context. The work of Li, et al.^32^ considered the organizational situational normality base factors such as situational normality and structural assurance. However, institutional theory and its corresponding three dimensions were not formerly proposed in this work^32^. Therefore, investigating trust in the organizational context based on institutional theory is still lacking in the literature.

### Institutional theory

Institutional theory has been widely applied in information systems research (shown in Table 3). Information technology (IT) adoption is the most frequently applied area for institutional theory, which has, for example, been used to explain the adoption behavior of interorganizational information systems^36^, grid computing^37^, e-government^38^ and open government data^39^. The second most frequently applied area for institutional theory is IT security. For example, institutional theory has been used to explain the behavior of information systems security innovations^40^, organizational actions for improving information systems security^41^, and data security policy compliance^42^. In addition, institutional theory has also been used in the knowledge-sharing context^43^ and IT strategy context^44^.

**Table 3:** Research on institutional theory in the information systems discipline.

Category	Context	Key variables	Source
IT adoption	Interorganizational linkage (financial electronic data interchange)	Mimetic, coercive, and normative pressures	^36^
	Grid computing	Mimetic pressures (social contagion), firm innovativeness, tendency to outsource, and IT department size	^37^
	E-government	Top management commitment, external institutional pressures	^38^
	Open government data	Existing institutional arrangements, internal and external institutional pressures	^39^
IT security	Information systems security innovations	Institutional conformity pressure, economic-based consideration	^40^
	Information systems security	Coercive, normative, and mimetic isomorphic processes	^41^
	Data security	Institutional and market forces	^42^
Knowledge sharing	Knowledge management systems	Institutional norms, trust	^43^
IT strategy	E-HRM	Regulative, cognitive and normative institutional dimensions	^44^

The literature review in Table 3 suggests that most studies based on institutional theory focus on observed behaviors (e.g., adoption behavior, innovations, security rule compliance, knowledge-sharing behavior) rather than psychological variables. According to institutional theory, institutional influence may also impact psychological variables such as trust. Furthermore, the linkage between institutional theory and trust has been validated by many studies. In the political science discipline, Heikkilä^44^ confirmed that formal political and legal institutions are positively related to generalized trust. Sønderskov and Dinesen^45^ suggested that institutional trust exerts a causal impact on social trust. In the information systems discipline, Chen and Wen^46^ found that people’s trust in AI is positively associated with institutional trust in government and corporations. Wang, et al.^43^ demonstrated that institutional norms have a positive influence on trust in the knowledge-sharing context. All the literature mentioned above suggests that linking institutional theory with trust is theoretically appropriate.

The literature review in Table 3 also suggests that most studies based on institutional theory examine only the organizational level. One exception is the work of Wang, et al.^43^, which investigates, at the individual level, how institutional norms may enhance knowledge sharing. The connotations of institutional theory imply that it can actually be applied at different levels, such as the organizational, group and individual levels^47^, or the federal, state and regional levels^42^. However, research on institutional theory at the nonorganizational level is still lacking. This study will thus contribute to the institutional theory literature by extending its conceptual dimensions to multiple levels (organizational level, group level and individual level).

## Hypotheses development

**Figure 1:**
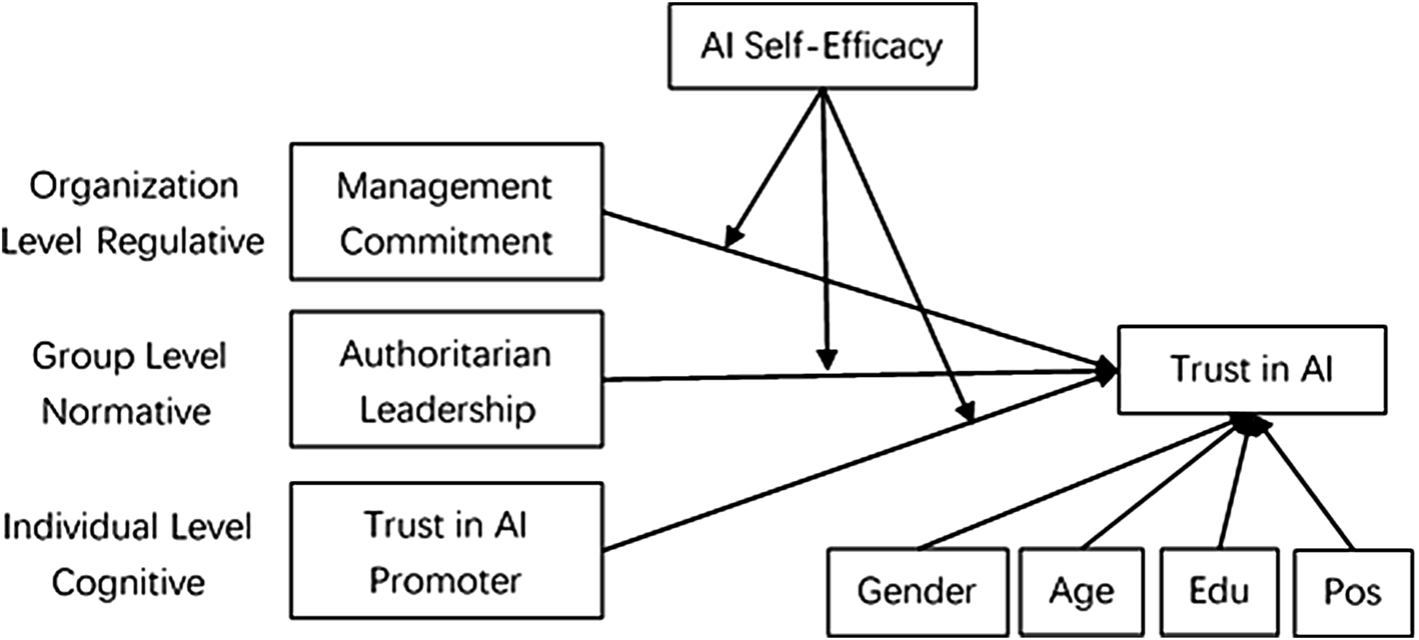
Research model.

The research model for this study is shown in Fig. 1. Three dimensions of institutional theory (regulative, normative and cognitive dimensions) are identified as antecedents of trust in AI. More specifically, management commitment, authoritarian leadership and trust in the AI promoter are hypothesized to have positive effects on trust in AI. Furthermore, personal trait AI self-efficacy is hypothesized to moderate the influence of these three institutional dimensions on trust in AI.

Institutional theory considers the processes by which structures, including schemes, rules, norms, and routines, become established as authoritative guidelines for social behavior^48^. According to institutional theory, organizational or individual decisions are not driven purely by rational goals of efficiency but also by institutional environments such as social and cultural factors^49^.

Institutional theory posits that there are three pillars of institutions: the regulative dimension, the normative dimension, and the cognitive dimension^50^. The regulative dimension of institutional theory corresponds to laws, regulations, contracts and their enforcement through mediation, arbitration or litigation^51^. The basis of the legitimacy of the regulative dimension is legally sanctioned. The normative dimension of institutional theory corresponds to the socially shared expectations of appropriate behavior and social exchange processes^51^. The basis of the legitimacy of the normative dimension is morally governed. The cognitive dimension of institutional theory corresponds to conceptual beliefs and mental models, scripts or conceptual frameworks to bridge differences in values or interests^51^. The basis of legitimacy of the cognitive dimension is conceptually correct.

Although institutional theory is often used to explain IT adoption behaviors^36,37^ or IT security behaviors^41,42^, it can also be used to explain the user’s psychological variables, such as trust^43^. Moreover, institutional theory can be applied at different levels^42,47^ because institutional theory is a multilevel construct^52^, and the influence of the institutional environment may thus originate from multiple levels, such as the organizational, the group, or the individual level. Therefore, we identify three levels of institutional environmental elements (management commitment at the organizational level, authoritarian leadership at the group level and trust in AI at the individual level) in this study.

Management commitment, which operates at the organizational level, corresponds to the regulative dimension of institutional theory. In this study, management commitment means the commitment of the company's top managers to the application of AI in the company. If the top managers have a strong will to use AI in the company, they will share their strategic vision, spend more resources and publish more incentivizing rules for it. For example, AI-related projects may receive more funding from top managers, and employees who are actively engaged in AI-related projects may receive more economic rewards and higher promotion opportunities. Support from the top manager acts as the endorsement to ensure that the AI is qualified and that the AI-related project will be successful. Therefore, higher management commitment to AI is associated with higher trust in AI. Consequently, we hypothesize that

H1: Management commitment is positively associated with trust in AI.

Authoritarian leadership, which operates at the group level, corresponds to the normative dimension of institutional theory. Authoritarian leadership is a leadership style characterized by domination and individual control over all decisions and little input from group members^53^. Authoritarian leadership is deeply rooted in the central Confucian thought of five cardinal relationships: the benevolent king with the loyal minister, the kind father with the filial child, the kind senior with the deferent junior, the gentle elder brother with the obedient younger brother, and the righteous husband with the submissive wife^53^. Therefore, authoritarian leadership that emphasizes authority, obedience, and unquestioning compliance is common in China^54^.

An authoritarian leader is encouraged to maintain absolute authority and require obedience. In groups with authoritarian culture, subordinates are required to obey the leader’s will without any question^54^. As a result, subordinates will check whether their ideas meet the leader’s expectations and alter them accordingly to avoid the leader's criticism or punishment. Since an AI project must be approved by the leader, subordinates will derive from such projects that the leader is inclined to trust AI. To obtain the leader’s favor, subordinates will aim to hold the same ideas as the leader. The attitude toward AI is no exception. Therefore, users in groups with high authoritarian culture are more inclined to trust in AI. Consequently, we hypothesize that

H2: Authoritarian leadership is positively associated with trust in AI.

Trust in the AI promoter, which operates at the individual level, corresponds to the cognitive dimension of institutional theory. In this study, AI promoters are the persons who are responsible for the introduction, implementation, user training and promotion of AI systems. They are similar to innovation champions but specific to the AI context. AI is a highly complicated black box for most users. It is quite difficult for ordinary users to understand the inherently complex mechanisms and far-reaching influence of AI. In contrast, it is easier for ordinary users to trust the AI promoter, for example, by considering whether AI promoters have sufficient professional expertise, whether they represent the company’s interests, or whether they will harm the individual’s interests. By considering these questions, ordinary users can decide whether they should trust the AI promoter. According to trust transfer theory, trust can be transferred when the target and the trusted party are contextually related^55^. If users trust the AI promoter, they will also trust the AI itself because it is backed by the AI promoter. Consequently, we hypothesize that

H3: Trust in the AI promoter is positively associated with trust in AI.

Self-efficacy refers to an individual's belief in his or her capacity to execute behaviors necessary to produce specific performance attainments^56^. In this study, AI self-efficacy refers to an individual's belief in his or her capacity to use or operate AI systems properly. AI self-efficacy moderates the influences of the three dimensions of institutional theory on trust in AI. First, different levels of AI self-efficacy reflect different levels of users' understanding of AI technology and also whether users can fully perceive the benefits to the company offered by AI. Users with higher AI self-efficacy are more likely to understand the benefits of AI for the company. Second, although the use of AI is beneficial for the company, it may also pose some challenges to individuals. For example, unfamiliarity with the operation of AI can result in certain learning costs or even potentially interfere with daily work. Users with higher AI self-efficacy are less likely to be concerned about the challenges posed by the AI system. In summary, users with higher AI self-efficacy perceive more benefit of AI for the company and fewer challenges posed by AI for the individual. As a consequence, the influences of the three institutional dimensions will be stronger for users with high self-efficacy. Consequently, we hypothesize that

H4: AI self-efficacy positively moderates (strengthens) the relationship between management commitment and trust in AI.

H5: AI self-efficacy positively moderates (strengthens) the relationship between authoritarian leadership and trust in AI.

H6: AI self-efficacy positively moderates (strengthens) the relationship between trust in the AI promoter and trust in AI.

## Research methodology

### Measurement

All the measurement items were adapted from existing validated scales (see Table 4). We slightly modified some items to ensure their suitability for our context. We used a seven-point Likert-type scale, ranging from 1 (“strongly disagree”) to 7 (“strongly agree”), to measure all items. Management commitment was measured using a four-item scale derived from Lewis, et al.^57^, which was originally used to measure management support that could influence information technology use in the organization. Authoritarian leadership was assessed with a six-item scale borrowed from Chen, et al.^58^, which was originally used to measure the leadership style in the Chinese context. Trust in the AI promoter was assessed with a four-item scale adapted from Kankanhalli, et al.^59^, which was originally used to measure the general good intent, competence, and reliability of other employees. AI self-efficacy was assessed with a two-item scale adapted from Venkatesh, et al.^60^, which was originally used to measure the user’s self-efficacy toward an information system. Trust in AI was assessed with a three-item scale adapted from Cyr, et al.^61^, which was originally used to measure the user’s trust toward a website.

**Table 4:** Measurement items.

Constructs	Items	Sources
Management commitment (MC)	The company is committed to a vision of using AI	^57^
	The company is committed to supporting AI-related projects	
	The company strongly encourages the use of AI	
	The company will recognize my efforts in AI-related projects	
Authoritarian leadership (AL)	My leader asks me to obey his/her instructions completely	^58^
	My leader always behaves in a commanding fashion in front of employees	
	My leader determines all decisions in the organization regardless of their importance	
	In my leader's mind, the standard subordinate is an employee who obeys his/her commands completely	
	We have to follow his/her rules to get things done. If not, he/she punishes us severely	
	My leader emphasizes that our group must have the best performance of all the units in the organization	
Trust in AI promoter (TP)	I believe that the AI promoters have sufficient expertise	^59^
	I believe that the AI promoters will put the company's interests first	
	I believe that the AI promoters will not harm my personal interests	
	I believe that the AI promoters will do their best to ensure the success of AI project	
AI self-efficacy (SE)	I could operate the AI system correctly if it provides guidelines or help manuals	^60^
	I could use the AI system correctly if I spent some time on it	
Trust in AI (TA)	I can trust the AI system	^61^
	I can trust the diagnosis made by the AI system	
	I will seriously consider the diagnosis made by this AI system	

### Data collection

To test the research model and hypothesis, we collected data through a survey conducted in a large petrochemical company in eastern China. The company has just launched an AI-based diagnostic system for fault detection and isolation in process equipment services. The system monitors the operation of equipment (e.g., rotating machinery), operates the deep learning algorithm in the background, and gives an alarm when it detects possible failure risks. Engineers check the equipment after receiving the alarm and decide whether further maintenance work is necessary in the future.

Manufacturing industries are those that engage in the transformation of goods, materials or substances into new products. The transformational process can be physical, chemical or mechanical. Discrete manufacturing and process manufacturing are two typical examples of manufacturing industry. Although discrete manufacturing and process manufacturing differ a lot, they are both involved in the use of machinery and industrial equipment. The AI-based diagnostics system for fault detection and isolation in equipment service should be applicable for both discrete and process manufacturing industry. Therefore, the selection of a petrochemical company is a representative example of manufacturing industry.

The engineers of the company are the ideal subjects for this study because they have some professional knowledge of and technical experience with equipment fault diagnostics. With the help of the company's technical management department, the survey was conducted from April 2020 through May 2020. A total of 206 engineers responded. After removing invalid or incomplete questionnaires, we obtained a total of 180 valid questionnaires. All experimental protocols were approved by the Ethics Committee in the School of Business, East China University of Science and Technology. All methods were carried out in accordance with relevant guidelines and regulations. Informed consent was obtained from all subjects or if subjects are under 18, from a parent and/or legal guardian.

Subjects’ demographic information is presented in Table 5. As noted in Table 5, the proportion of males (88.3%) was much higher than that of females (16.7%). This disparity is because males usually account for the vast majority of production-oriented petrochemical company employees. We confirmed that the ratio of males to females in Table 5 was consistent with the actual ratio of employees.

**Table 5:** Respondent demographics (*n* = 180).

	Item	Percentage
Gender	Male	88.3
	Female	16.7
Age	≤ 30	10
	31–40	56.1
	41–50	23.3
	≥ 51	10.6
Education	Junior college and below	11.7
	Undergraduate	65.0
	Master’s	22.2
	Ph.D.	1.1
Position	Assistant Engineer	18.9
	Engineer	51.1
	Senior Engineer	30.0
	Professorate Senior Engineer	6.7

Because the data were collected from a single source at the same time and were perceptual, we further tested for common method bias. We followed Harman’s single-factor method^62^ to evaluate the five conceptual variables in our model. The first factor accounted for 36.49% of the variance. Therefore, the threat of common method bias for the results was minimal.

## Analysis and results

Partial least squares (PLS) was used to test the research model and hypothesis. We used smartPLS Version 2.0 in our analysis.

### Measurement model

To ensure the validity of the research conclusion, we need to check that the constructs provided in the research model were correctly measured by the scale items in the questionnaire. Validity and reliability are two key factors to consider when developing and testing any survey instrument. Validity is about measurement accuracy, while reliability is about the measurement of internal consistency. Therefore, the measurement model was evaluated by testing construct validity and reliability.

To test convergent validity, we examined the loadings and average variance extracted (AVE). As shown in Table 6, the loadings of all items except one were above the cutoff value of 0.7. For the fifth item of authoritarian leadership, the loading value is 0.674. Since 0.674 is very close to 0.7, and the six-item measure of authoritarian leadership was borrowed from a single study^58^, we included all six items of authoritarian leadership in the following analysis to ensure that the concept was completely covered. The AVE values ranged from 0.685 to 0.930, above the desired value of 0.5. All these results demonstrated the adequate convergent validity of the measurement model^63,64^.

**Table 6:** Results of confirmatory factor analysis.

Construct	Items	Loading	Cronbach’s alpha	Composite reliability	Average variance extracted
Management commitment (MC)	4	0.894	0.930	0.950	0.827
		0.913			
		0.939			
		0.890			
Authoritarian leadership (AL)	6	0.853	0.894	0.897	0.594
		0.850			
		0.713			
		0.726			
		0.674			
		0.791			
Trust in AI promoter (TP)	4	0.885	0.884	0.930	0.742
		0.876			
		0.812			
		0.870			
AI self-efficacy (SE)	2	0.922	0.785	0.902	0.822
		0.891			
Trust in AI (TA)	3	0.895	0.852	0.910	0.772
		0.886			
		0.853			

To test construct reliability, we focused on Cronbach's alpha and composite reliability^65^. As shown in Table 6, the minimum of Cronbach's alpha was 0.785, which was higher than the recommended value of 0.7. The minimum value of composite reliability was 0.897, which was also higher than the recommended value of 0.7. The results of Cronbach's alpha and composite reliability indicated that our constructs had no problem in reliability.

To test the discriminant validity, we compared the correlations among constructs and the square root of AVE^65^. As shown in Table 7, the correlation coefficients among constructs were between 0.000 and 0.640, which were lower than the recommended value of 0.71^66^. Meanwhile, the square roots of the AVEs (shown on the diagonal of Table 7) were greater than the corresponding correlation coefficients underneath. The results in Table 7 showed that our measurement model had good discriminant validity.

**Table 7:** Means, standard deviation and correlation.

	Mean	SD	MC	AL	TP	SE	TA	Gen.	Age	Edu	Pos.
MC	5.544	1.225	**0.909**								
AL	4.500	1.773	0.109	**0.771**							
TP	5.647	0.978	0.589	0.151	**0.861**						
SE	5.711	0.938	0.450	0.228	0.562	**0.907**					
TA	5.535	0.749	0.498	0.218	0.640	0.365	**0.879**				
Gender	0.833	0.140	0.000	− 0.101	− 0.063	0.017	− 0.094	**NA**			
Age	2.344	0.640	0.140	0.088	0.128	0.148	0.075	− 0.156	**NA**		
Education	2.139	0.366	− 0.015	− 0.088	0.006	− 0.031	0.007	0.144	− 0.203	**NA**	
Position	2.122	0.477	0.131	− 0.054	0.100	0.136	0.108	− 0.014	0.277	0.387	**NA**

NA: not applicable. The square root of AVE is the bold numbers in the diagonal row.

### Structural model

We compared the five models hierarchically (as shown in Table 8). In Model 1, only the control variables were included. The independent variables were added in Model 2. In Models 3 through 5, the interaction terms of the independent variables were added.

**Table 8:** Stepwise PLS results.

Variables	Model 1	Model 2	Model 3	Model 4	Model 5
Gender	− 0.091	− 0.052	− 0.040	− 0.048	− 0.034
Age	0.053	− 0.050	− 0.066	− 0.058	− 0.066
Education	− 0.012	− 0.009	− 0.040	− 0.015	− 0.030
Position	0.113	0.060	0.089	0.082	0.080
MC (management commitment)		0.192**	0.190**	0.201**	0.176**
AL (authoritarian leadership)		0.129*	0.173**	0.115*	0.173**
TP (Trust in AI promoter)		0.532***	0.568***	0.570***	0.595***
SE (AI self-efficacy)		− 0.049	− 0.916*	− 0.434	− 0.830
MC*SE			0.870*		
AL*SE				0.378	
TP*SE					0.783*
R^2^	0.029	0.453	0.510	0.464	0.514

*p < 0.05; **p < 0.01; ***p < 0.001.

The results of Model 1 showed that the four factors of gender, age, education and position explained 2.9% of the variance of the dependent variable. All four control variables have no significant impacts on Trust in AI.

The results of Model 2 showed that management commitment (β = 0.192, p < 0.01), authoritarian leadership (β = 0.129, p < 0.05) and trust in the AI promoter (β = 0.532, p < 0.001) were significantly related to trust in AI. Therefore, H1, H2 and H3 were all supported. The results indicate that strong leadership at the institutional level is crucial to promote trust in AI (H1), strong advocacy at the mid-management level has a positive impact on trust in AI (H2) and trust in AI promoter at the individual worker’s level supports trust in AI (H3).

**Figure 2:**
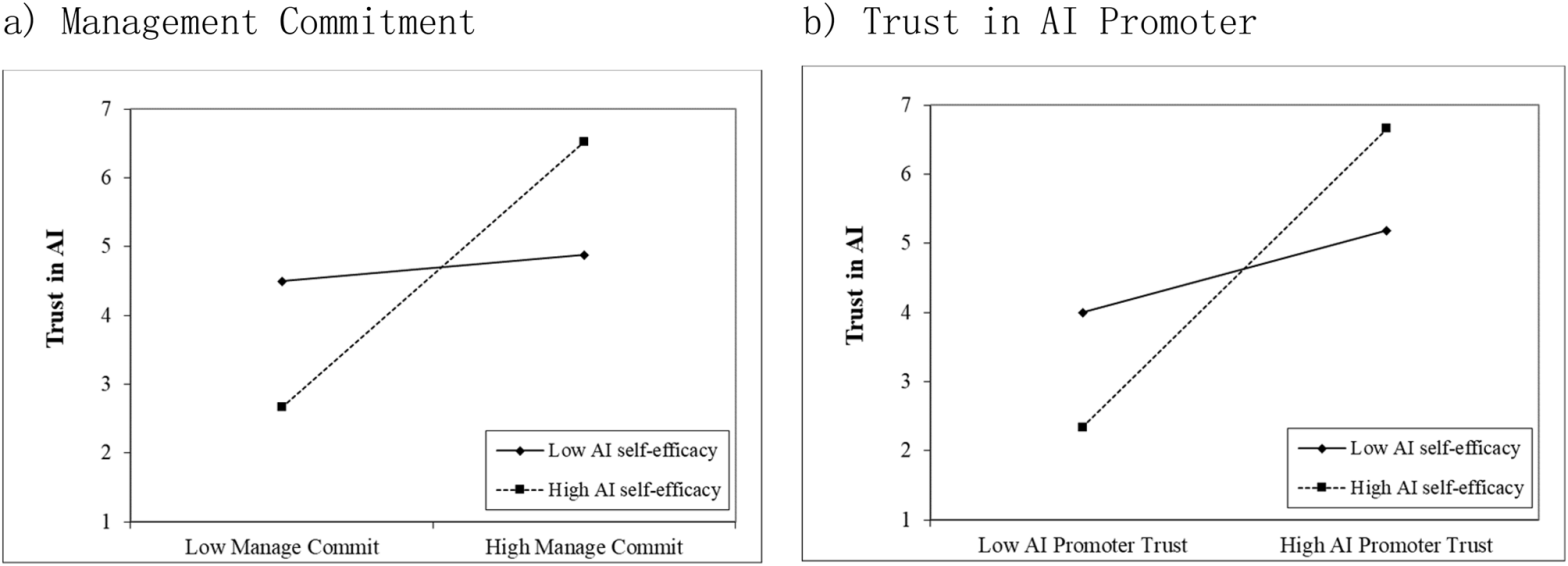
The moderating effect of AI self-efficacy on (**a**) management commitment and (**b**) trust in AI promoter.

The results of Model 4 showed that there was a significant positive interaction between management commitment and AI self-efficacy (β = 0.870, p < 0.05). The interaction plot between management commitment and AI self-efficacy (shown in Fig. 2a) suggests that the effect of management commitment is strengthened by AI self-efficacy. Therefore, H4 is supported. The results of Model 5 showed that the interaction between authoritarian leadership and AI self-efficacy was not significant (β = 0.378, p > 0.05). Therefore, H5 was not supported. The results of Model 6 showed that there was a significant positive interaction between trust in the AI promoter and AI self-efficacy (β = 0.783, p < 0.05). The interaction plot between trust in the AI promoter and AI self-efficacy (shown in Fig. 2b) suggests that the effect of trust in AI is strengthened by AI self-efficacy. Therefore, H6 is supported.

## Discussion and Implications

### Major findings

Several major findings were obtained in this study. First, management commitment is positively associated with trust in AI. This finding implies that the support from the top manager acts as the endorsement to ensure that the AI is qualified and that the AI-related project will be successful.

Second, authoritarian leadership is positively associated with trust in AI. This finding implies that subordinates in groups with authoritarian culture are more inclined to trust in AI to maintain continuity of ideas with their leader.

Third, trust in the AI promoter is positively associated with trust in AI. This finding implies that although ordinary users may have difficulty understanding AI as a black box, they can turn to trust in the AI promoter. Trust in AI promoters can be transferred to trust in AI itself.

Fourth, AI self-efficacy is found to positively moderate the relationship between management commitment and trust in AI, as well as the relationship between trust in the AI promoter and trust in AI. This finding implies that for users with high AI self-efficacy, the impact of management commitment and trust in AI on overall trust in AI is higher than that for users with low AI self-efficacy. This occurs because users with high AI self-efficacy can perceive more benefit of AI for the company and fewer challenges posed by AI for the individual.

Fifth, the moderating effect of AI self-efficacy on authoritarian leadership is not significant. One possible explanation is that an authoritarian leader may ask subordinates to trust in AI directly, and any disobedience will lead to punishment. As a result, subordinates will always express trust in AI regardless of how much they benefit or how many challenges they perceive from it. This finding is different from the assumption in the hypothesis development phase. In the hypothesis development section, we assume that subordinates speculate that the leader's attitude toward AI is positive. However, we do not assume that the leader will ask the subordinates to trust in AI directly.

### Practical implications

This study provides some valuable guidelines for practitioners. First, our study suggests that support from top managers is important for trust in AI in the manufacturing industry. Users observe the regulative institutional elements (e.g., strategic vision, resource allocation and incentive rules) for AI projects to infer the quality of AI and success probability of AI projects. Therefore, top managers should send positive signals to employees about the company’s commitment to supporting AI.

Second, our results indicate that users in authoritarian organizational culture are more inclined to trust in AI. Although the effect of authoritarian leadership is still controversial, this paper proves that users in authoritarian culture are more willing to trust in AI. Therefore, managers should understand that groups with authoritarian culture have more advantages in using AI in the manufacturing industry.

Third, our study suggests that AI promoters are very important to building trust in AI for ordinary users. Since users cannot understand the black box of AI itself, they decide whether they should trust the AI promoter. Therefore, managers should select AI promoters carefully and ensure that they will be trusted by ordinary users.

Fourth, we suggest that the effects of institutional dimensions depend on the user’s AI self-efficacy. The impacts of management commitment and trust in the AI promoter will be more salient for users with high AI self-efficacy. Therefore, managers should try to promote employees’ AI self-efficacy. For example, managers can hold AI training classes, organize employee viewings of AI science and education films, or provide trials of simple AI programs to improve employees' understanding of AI.

### Limitations

This study has some limitations that open up avenues for future research. First, all the variables used in this study contain only self-reported data. Although it is very difficult to collect some objective data in the initial stage of an AI program, we intend to include some objective data, such as the interaction patterns and usage patterns, to better explain trust in AI in future work.

Second, the task type and explanation design elements of AI are not considered in this study. The user’s trust in AI may depend on the task type and design elements that support the task. For example, users may have different tendencies to trust in AI for simple tasks and complex tasks. Different types of explanations (e.g., mechanism explanations, case explanations, case comparisons) may also have different effects on users’ trust in AI. The influence of task type and explanation mechanism of AI should be considered in the future.

Third, this research focuses only on a fairly small group of employees coming from a single company and a specific geographical location. Therefore, the findings cannot be generalized at this point. This study should be extended to other companies at different levels of production and technological advancement, as well as other companies with different geographical locations and different cultural habits in the future. Other types of manufacturing (e.g., discrete manufacturing) companies should also be investigated to increase the generalizability of research findings.

Fourth, the survey subjects used in this study are from a company that just launched an AI-based diagnostic system. Therefore, the research findings about this study can only be applied to the initial trust toward AI. As users have more interactions with the AI system and get more feedbacks about the correctness of output, their trust toward AI may be more influenced by their individual experiences. Therefore, it must be cautious to generalize the findings of this paper to users with a longer AI system experience.

Last, the research was conducted in the Chinese context and authoritarian leadership was found to play an essential role in promoting trust in AI. However, the authoritarian leadership characterized as obedience and unquestioning compliances may not be accepted as guiding principles for societies with other cultural backgrounds. Therefore, the practical implications of authoritarian leadership may be greatly reduced for the individualistic cultures.

## Conclusion

This study investigates trust in AI in the manufacturing industry from an institutional perspective. We identified three institutional dimensions from institutional theory and conceptualized them as management commitment, authoritarian leadership, and trust in the AI promoter. We hypothesized that all three institutional dimensions have positive effects on trust in AI. In addition, we hypothesized the moderating effects of AI self-efficacy on three institutional dimensions. A survey was conducted in a large petrochemical enterprise in eastern China just after the company had launched an AI-based diagnostics system for fault detection and isolation in process equipment service. The results indicate that management commitment, authoritarian leadership, and trust in the AI promoter are all positively related to trust in AI. Moreover, the effect of management commitment and trust in the AI promoter are strengthened when users have high AI self-efficacy. The findings of this study provide suggestions for academics and managers in promoting users’ trust in AI in the manufacturing industry.
